# Multilocus Sequence Typing (MLST) and Random Polymorphic DNA (RAPD) Comparisons of Geographic Isolates of *Neoparamoeba perurans*, the Causative Agent of Amoebic Gill Disease

**DOI:** 10.3390/pathogens8040244

**Published:** 2019-11-19

**Authors:** Jessica C. Johnson-Mackinnon, Philip B. B. Crosbie, Egil Karlsbakk, Mar Marcos-Lopez, Richard Paley, Barbara F. Nowak, Andrew R. Bridle

**Affiliations:** 1 Institute for Marine and Antarctic Studies, University of Tasmania, Locked Bag 1370 Launceston, Tasmania 7250, Australia; philip.crosbie@utas.edu.au (P.B.B.C.); Andrew.Bridle@utas.edu.au (A.R.B.); 2 Department of Biology, University of Bergen, N5020 Bergen, Norway; egil.karlsbakk@uib.no; 3 Vet-Aqua International, Unit 7B, Oranmore Business Park, H91 XP3F Galway, Ireland; mar.marcos-lopez@fishvetgroup.com; 4 Centre for Environment Fisheries and Aquaculture Science (Cefas), Weymouth laboratories, The Nothe Barrack Road, Weymouth, Dorset DT4 8UB, UK; richard.paley@cefas.co.uk

**Keywords:** *Paramoeba perurans*, Amoebic Gill Disease, epidemiology, pathogen genetics

## Abstract

*Neoparamoba perurans*, is the aetiological agent of amoebic gill disease (AGD), a disease that affects farmed Atlantic salmon worldwide. Multilocus sequence typing (MLST) and Random Amplified Polymorphic DNA (RAPD) are PCR-based typing methods that allow for the highly reproducible genetic analysis of population structure within microbial species. To the best of our knowledge, this study represents the first use of these typing methods applied to *N. perurans* with the objective of distinguishing geographical isolates. These analyses were applied to a total of 16 isolates from Australia, Canada, Ireland, Scotland, Norway, and the USA. All the samples from Australia came from farm sites on the island state of Tasmania. Genetic polymorphism among isolates was more evident from the RAPD analysis compared to the MLST that used conserved housekeeping genes. Both techniques consistently identified that isolates of *N. perurans* from Tasmania, Australia were more similar to each other than to the isolates from other countries. While genetic differences were identified between geographical isolates, a BURST analysis provided no evidence of a founder genotype. This suggests that emerging outbreaks of AGD are not due to rapid translocation of this important salmonid pathogen from the same area.

## 1. Introduction

Amoebic Gill Disease (AGD) is an emerging disease which is becoming one of many health concerns for global salmon aquaculture and is caused by the ubiquitous marine amoeba *Neoparamoeba perurans* [1,2]. The first documented cases of AGD occurred in the mid-1980s and for many years the disease predominantly affected Atlantic salmon cultured in Tasmania, Australia [3] and the USA [4]. More recently, the frequency and distribution of AGD outbreaks have increased to include every major salmon producing country except for Iceland [5]. Outbreaks were also reported in Spain and South Africa, but farming of Atlantic salmon was discontinued in both locations [5]. At present, there are reports of AGD outbreaks in thirteen countries across six continents and there is evidence that *N. perurans* DNA is present in regions where outbreaks have not yet occurred. Amoebae from the genus *Neoparamoeba* are presumed to be ubiquitous in the marine environment [6,7,8].

*N. perurans* was confirmed as the aetiological agent of AGD by fulfilling Koch’s postulates [2]. It was first described and identified as the only species of amoeba present in histological sections of AGD affected gills of Atlantic salmon [1] when the *18S rRNA* gene was successfully used to differentiate between the three species from genus *Neoparamoeba.* The *18S rRNA* gene appears to lack the polymorphic variation to differentiate *N. perurans* isolates [9]. Conversely, another common phylogenetic marker, the internal transcribed spacer (ITS) genes, appeared too polymorphic to characterise *N. perurans* isolates with intra-genomic length differences, making sequencing difficult [10]. There are apparent intra-genomic differences in ITS regions within clones of closely related sister species *N. pemaquidensis* [11], and there is evidence of this within the same regions in *N. perurans* [10]. An ideal typing method, in this case, would be sensitive enough to detect different populations of *N. perurans* but not so sensitive as to detect differences between individuals. Therefore, other methods are required to differentiate between geographic isolates of the same species.

Multilocus Sequence Typing (MLST) is a PCR-based technique that compares gene sequences from several loci, based on the number of nucleotide differences per allele per gene. The method was first described in a study on the bacterium *Neisseria meningitis* [12]. Since then, the technique has been successfully used to reveal genetic diversity in prokaryotic populations [13,14]. MLST has also been applied to a growing variety of eukaryotic organisms as a typing method, including other parasitic organisms such as *Leishmania* spp., *Trypanosoma cruzi, Entamoebae histolytica* and *Acanthamoeba* spp. [15,16,17,18,19]. However, applying MLST to eukaryotes is more difficult due to the diploid or polyploid nature of these organisms [20] and, as a consequence, issues with handling heterozygous or multi-state sites are generally treated as ambiguous information and ignored [20]. Despite this, a classical MLST analysis was applied to the eukaryotic species and was successful in resolving differences between isolates for *Entamoeba* and *Acanthamoeba*.

Random Amplified Polymorphic DNA (RAPD) was first developed by Williams et al. [21] as an alternative to Random Fragment Length Polymorphisms (RFLP) for the creation of genetic maps. RAPD assays rely on a number of short oligonucleotide (8–12 bp) arbitrary primers that lack palindromic sequences and have a high CG content [22]. There is a high probability that most genomes contain several small inverted repeats close together. When amplified through PCR, the short RAPD primers bind to these small inverted repeats within the genome and amplify the intervening DNA segments, producing profiles of bands of different size when visualized on a gel [23]. As a genotyping technique, RAPD has gained popularity due to several factors: (a) there is no need for prior genomic sequencing information, which makes it very useful in non-model organisms, (b) it is a low cost relative to other molecular methods, and (c) it is relatively quick for determining genetic differences between and within species [22,24].

RAPD is a useful tool for when no defining phenotypic traits exist [21] as in the *Neoparamoeba* genus. RAPD has been successful in a variety of free living and opportunistic amoebae, including *Naegleria, Entamoeba* and *Acanthamoeba* [18,25,26]. The genus *Acanthamoeba* contains free living amoebae that are opportunistic parasites to a variety of species, including humans [26]. In a study comparing 11 Brazilian isolates with eight American reference isolates, the 11 Brazilian isolates were successfully grouped separately to the eight American strains using RAPD [26].

Both MLST and RAPD can be useful in improving our understanding of the epidemiology of infections such as AGD. This is important, as AGD outbreaks have been steadily increasing and have a larger geographic distribution, occurring in thirteen countries across six continents, compared to the initial outbreak in Atlantic salmon in Tasmania [3,5,8]. Whole nuclear genome sequence information is currently lacking for *N. perurans*, with only partial sequence data from transcriptome analyses publicly available [9,10]. In the absence of whole genome analysis, MLST or RAPD schemes offer valuable insight into the global epidemiology of this significant pathogen. The RAPD analysis was chosen as there is little sequencing data available for *N. perurans* and the results can be used to help inform the MLST analysis and create the groundwork for further studies.

In this study, MLST and RAPD were applied to a variety of environmental and cultured (clonal and non-clonal) *N. perurans* isolates from Canada, Ireland, Norway, Scotland, Tasmania, and the USA, to examine if *N. perurans* isolates form phylogenetic groupings based on their geographic origin. If they do, then it is likely that transmission is a natural occurrence rather than an unintentional transmission. The significance of our research is the characterisation of geographical isolates of *N. perurans* as the causative agent of an emerging disease affecting salmon farming worldwide.

## 2. Results

### 2.1. PCR Amplification and Sequencing

PCR amplification of six gene fragments (elongation factor 1, elongation factor 2, RNA polymerase large subunit 1, beta actin, beta tubulin and succinate dehydrogenase complex flavoprotein subunit A) was applied to all 16 isolates. The sequences are reported in Genbank (accession numbers KX363875–KX363883). Sense and antisense strands were sequenced for the gene amplicons across all 16 isolates, with the exception of elongation factor 1 (*ef1*). For *ef1*, the antisense strand could not be sequenced from two isolates—Ireland 1 and Norway—due to insufficient material for these isolates, caused by low DNA concentration and poor quality in some isolates. Because of this, only the sense strand sequences were used for this gene in MLST analysis.

### 2.2. MLST Analysis

**Figure 1:**
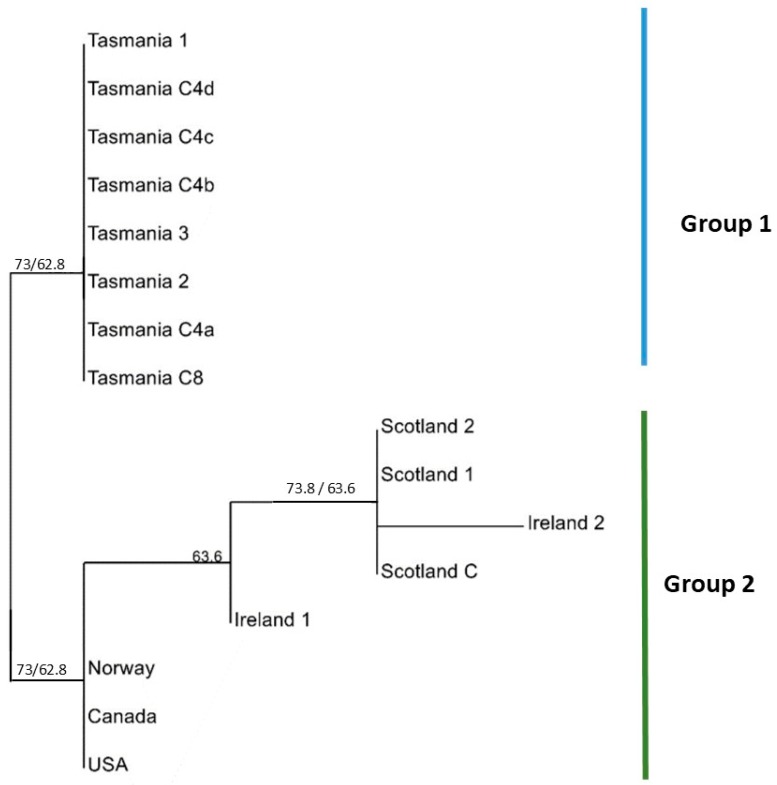
Concatenated Neighbour Joining Tree based on six Multilocus Sequence Typing (MLST) gene loci (elongation factor 1 (*ef1*), beta tubulin (*tubb*), RNA polymerase large subunit 1 (*Rpb1*), beta actin (*actb*), elongation factor 2 (*ef2*) and succinate dehydrogenase complex flavoprotein subunit A (*sdha*) using average states to resolve polymorphic sites. Different groupings are represented by vertical bars. Two distinct groups can be visualized; Group 1 (Tasmania) and Group 2 (Ireland, Scotland, the USA, Canada and Norway). Branch support represents bootstrap values (1000 replications). Blue values represent the bootstrap support for the SNP duplication method (1000 replications).

The MLST analysis using concatenated sequences and a neighbour joining tree identified the Tasmanian isolates as grouping separately from all other isolates (Figure 1). Within the non-Tasmanian group, Ireland and Scotland were grouped together, as were Norway, Canada and the USA. For the concatenated neighbour joining trees generated for all six loci, the bootstrap values were slightly higher when using the ‘single nucleotide polymorphism (SNP) duplication’ method compared to the ‘average states’ method. When the ‘average states’ was applied, the Tasmanian isolates grouped separately to all other geographic isolates with a bootstrap of 62.8%. Ireland and Scotland were together as a subgroup of the Northern Hemisphere isolates, with 63.6% bootstrap support (Figure 1). The same tree topology could be observed when concatenated neighbour joining tress were generated using SNP duplication instead of average states, however, bootstrap support changed, with the subgroup (Ireland and Scotland) having 73.8% support, and the Northern Hemisphere group having 73% bootstrap support (Figure 1 values in blue). Though the two topologies were identical, the Incongruence Length Difference (ILD) was significant for the SNP duplication method (P = 0.0009), whereas it was not significant using the average states method (P = 1). This, combined with the fact that SNP duplication resolves differences by duplicating the bases and thus modifying the alignment, which could have methodologically altered the bootstrap values, meant that the average states method was chosen as the most appropriate.

Table 1 shows the allelic profile of the six loci analysed by the MLSTest software. There were very few polymorphic sites reported, with the maximum for any given gene locus being one. The least discriminatory power was shown by *ef1*, *Rpb1* and *sdha* (0.118), with one genotype and no polymorphic sites. Βeta actin (*actb)* had the highest discriminatory power (0.588) though not the highest number of genotypes (*ef2)*.

### 2.3. BURST Analysis

**Figure 2:**
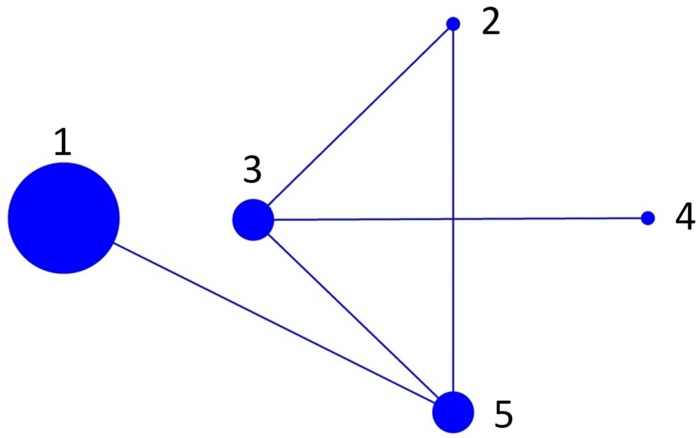
BURST graph of all sequences used in the MLST analysis. There were five sequence types defined: 1 (Tasmanian isolates), 2 (Ireland 1), 3 (Scotland Isolates), 4 (Ireland 2) and 5 (USA, Canada and Norway). The graph shows a connection between all isolates typical of a clonal BURST configuration.

BURST analysis resolved five sequence types (ST): 1 (all isolates from Tasmania, including clones), 2 (Ireland 1), 3 (all isolates from Scotland), 4 (Ireland 2) and 5 (USA, Canada and Norway). All the sequence types were related to each other and there was no predicted founder (Figure 2).

### 2.4. RAPD Analysis

**Figure 3:**
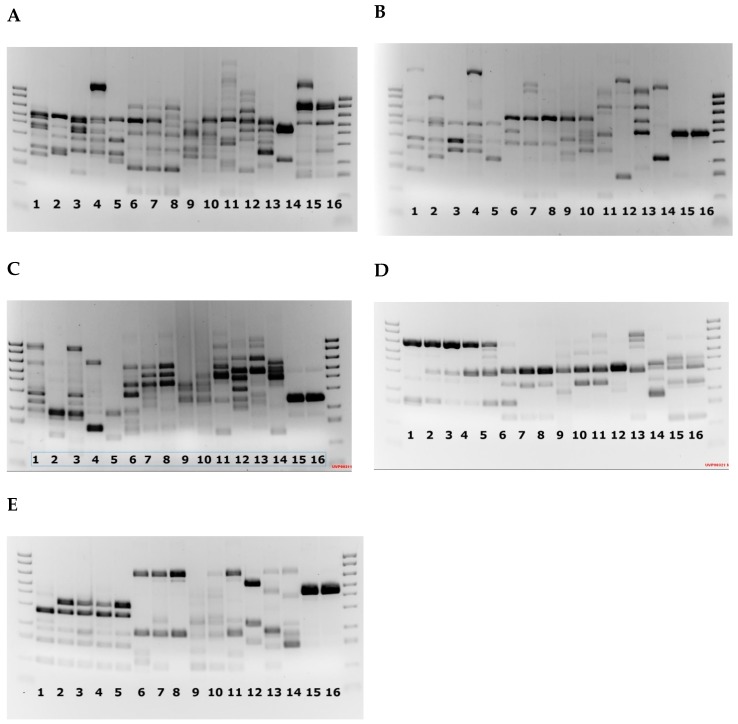
(**A**–**E**) Agarose gel of amplified Random Polymorphic DNA (RAPD) products for primers A1, A15, B10, B12 and B18, respectively. Lane 1, Tasmanian clone C8a; lane 2, Tasmanian clone C4a; lane 3, Tasmanian clone C4b; lane 4, Tasmanian clone C4c; lane 5, Tasmanian clone C4d; lane 6, Tasmanian isolate 1; lane 7, Tasmanian isolate 2; lane 8, Tasmanian isolate 3; lane 9, Norway; lane 10, Canada; lane 11, USA; lane 12, Ireland2; lane 13, Ireland1; lane 14, Scotland clonal isolate; lane 15, Scotland isolate 1 and lane 16, Scotland isolate 2.

The RAPD profiles across all five primers were highly polymorphic with consistent and reproducible banding patterns across all replicates (Figure 3). A total of 81 scorable bands were generated from all five primers.

**Figure 4:**
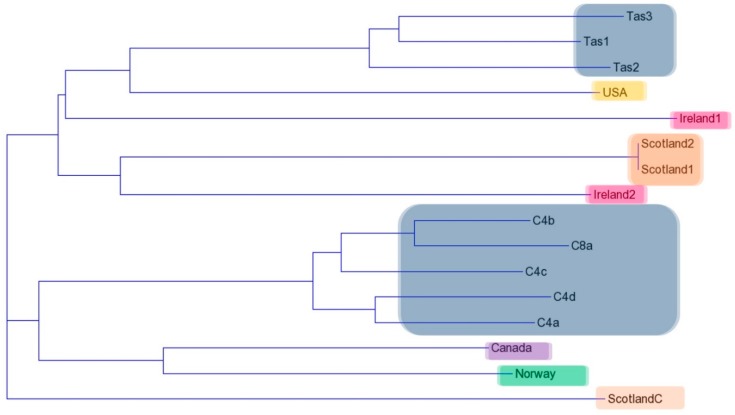
Dendrogram created from the Jaccard coefficient distance matrix for the combined RAPD primer profiles (A1, A15, B10, B12 and B18). The colour blocks represent the geographic locations of each isolate (Australia—grey, Scotland—brown, Ireland—pink, USA—orange, Canada—purple and Norway—green).

There was a high percentage of similarity between all three replicate runs using the RAPD primers. Only bands that were present in all three runs were included in the Jaccard coefficient analysis. Results from the RAPD analyses with all five primers were combined and a Jaccard coefficient was used to calculate a dissimilarity distance matrix (Table 2). The dissimilarity matrix was then inputted into the Trex—online web program (http://www.trex.uqam.ca/) under the neighbour joining option taken from Saitou and Nei [27], and a dendrogram was produced. There were several distinct groupings visualized with all Australian wild isolates grouped together and a separate grouping of all Australian clonal isolates. The Scottish non-clonal isolates were grouped together without the clonal isolate (Figure 4).

**Figure 5:**
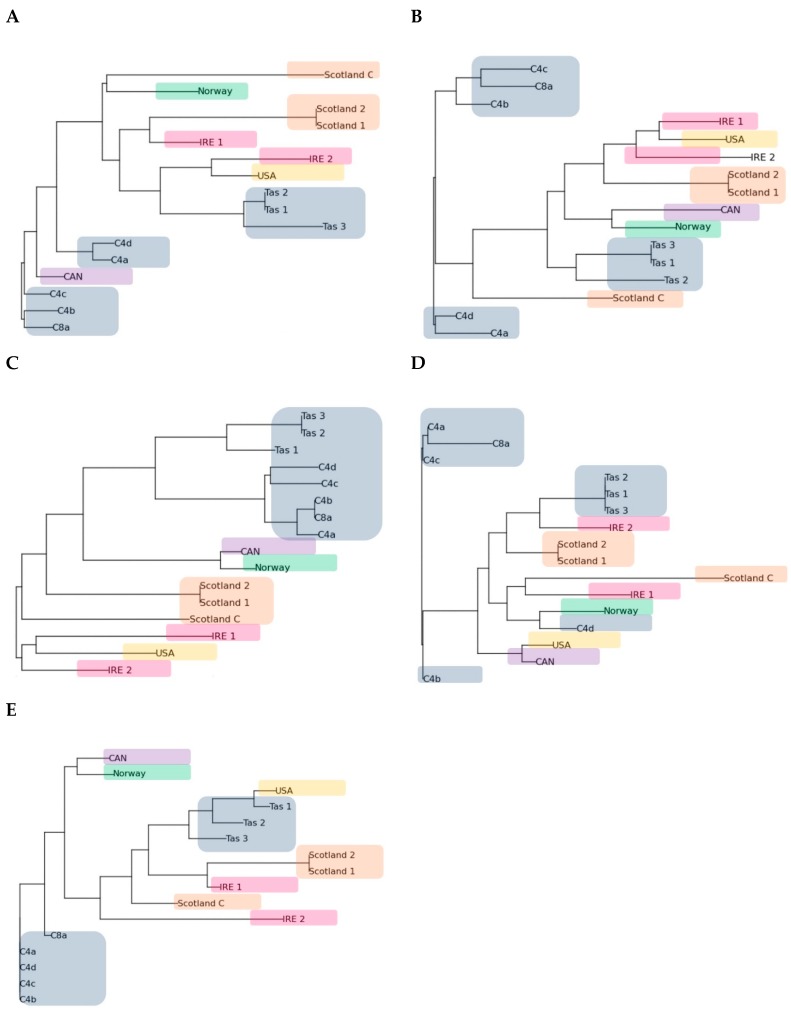
(**A**–**E**) Dendrograms of the N. *perurans* isolates created using the neighbour joining clustering in the PyElph program for RAPD primes A1, A15, B10, B12 and B18, respectively. Colours represent geographic locations (Australia—grey, Scotland—brown, Ireland—pink, USA—orange, Canada—purple and Norway—green).

The dendrograms created for each primer showed variation across isolates and primers with a few geographical consistencies (Figure 5). Primer B10 showed the highest level of geographic grouping with all Australian isolates as well as all Scottish Isolates grouping together. Norway and Canada grouped together when primers A15, B10 and B18 were used, and the Scottish and Irish isolates grouped together with some isolate variation in every primer dendrogram.

### 2.5. Assessment of Potential Bacterial Contamination

Due to the nature of the received isolates, it was impossible to ensure that no bacterial contamination was present. In previous work with RAPD, it was demonstrated that for contaminating DNA to have an effect on the RAPD analysis it would have to be in large proportions within the sample [28,29]. To investigate if bacterial contamination might have influenced the MLST or RAPD analysis, DNA from isolates were qPCR tested using universal bacterial *16S rRNA* gene, 27F and 518R primers and found to have low to trace amounts, only detectable after 33–40 cycles by qPCR. Therefore, combined with the inherent species specificity of the MLST primers, bacterial contamination was deemed insignificant. A further confirmatory check was performed using bacteria isolated from the supernatant of a laboratory *N. perurans* culture that had at least two orders of magnitude more bacteria than detected in the geographic isolates as bacterial equivalents using qPCR. This quantity of bacterial DNA failed to produce any detectable amplicon using the amoebae-specific MLST primers. Similarly, the same amount of DNA from this same bacterial isolation did not produce a detectable banding pattern on an agarose gel when RAPD analysis was performed using the five RAPD primers.

## 3. Discussion

This study represents an attempt to quantify genetic differences between *N. perurans* isolates using two molecular methods, MLST and RAPD. Based on the MLST analysis, there were minor sequence differences between geographically distinct isolates. All Tasmanian isolates grouped together and were separate from the rest of the isolates in the dendrogram. This is not surprising, given that they were from the geographical region separated by large distances and environmental barriers to the remaining locations. There were no sequence differences observed between the clonal and non-clonal isolates within the Tasmanian grouping across all six genes. Within the second group, there were limited substitutions to differentiate between isolates; nonetheless, the analysis resolved the Irish and Scottish isolates grouping together, with sequence variation in the Irish isolates. While Irish and Scottish isolates were in culture for some time, samples from the USA and Canada were from a direct isolation from Atlantic salmon gills and the sample from Norway was from a gill fixed in ethanol, so none of those three samples were cultured.

The analysis showed relatively low bootstrap support. Lower bootstrap support is expected with short sequences and few polymorphisms [30]. Shorter sequence lengths are known to reduce bootstrap support, for example, in a barcoding analysis in fungi reducing the sequence length caused the bootstrap support to fall below the significance cut-off, while maintaining the same tree topology [31].

The small number of observed differences may be partly due to *N. perurans* being a marine microorganism. It has been postulated that the sheer number of individuals in any given microbial species is so large that dispersal would rarely be restricted by contrived geographical barriers [32]. This postulation is amplified further when considering that *N. perurans* has few obstacles to geographic dispersal [5]. For example, certain species of foraminifera (marine protozoa) have genetically identical isolates collected from locations as separate as the Arctic and Antarctic [32]. Based on this theory of dispersion, the number of polymorphisms in any given *N. perurans* housekeeping gene is expected to be low.

Unlike the more studied genera of amoeba *Acanthamoeba* and *Entamoeba*, which tend to have more defined and constrained dispersal routes, the MLST analysis for *N. perurans* did not reveal fine scale resolution of population structure [13,18]. When the BURST analysis was considered, the evidence for highly genetically different geographic populations based on changes in conserved genes was further diminished. The BURST graph showed connections between all sequences and did not predict a primary founder. A primary founder denotes the genotype from which all subsequent genotype populations have descended [33]. This lack of a primary founder is to be expected within ubiquitous asexually reproducing populations. On a global scale, there appears to be no “source” population to which subsequent specific geographic outbreak populations can be traced. Therefore, this study suggests that the *N. perurans* from geographically separate AGD cases are not linked to the spread of *N. perurans* strains parasitic to fish from one initial outbreak site. Extensive surveys of wild fish species, including salmonids such chinook and coho salmon, returned no evidence of *N. perurans*, making wild fish transmission unlikely [5]. The trend of increasing outbreaks is more likely to be correlated with changes in environmental conditions, such as increasing global sea surface temperatures, or with intensification of aquaculture and the development of fish parasitic strains on a regional scale [8,34].

The new gene sequence data generated in this study remain largely uncharacterized. Nevertheless, it should be noted that basic confirmatory sequence analysis was undertaken. All of the *N. perurans* isolates, whether clonal or not, were tested by specific 18S PCR for presence of known potential contaminating related amoebae, and none were detected. Furthermore, in the isolates used to generate the target sequence information, no other contaminating eukaryotic microbes were observed. The amplifying primers for the target genes were initially designed from the limited sequence information of related amoebae available in the databases—in large part from significant genomic data for *Neoparamoeba pemaquidensis*. When compared against the sequence databases by BlastN analysis, the de novo sequences generated from *N. perurans* isolates showed from 88% to 98% sequence identity over 100% of the query coverage to either previous established *N. perurans* (*actb*) or *N. pemaquidensis* (*actb, tubb, ef2, Rpb1*) or *N. branchiophila* (*ef1*) sequences. The next nearest second hit typically showed 10% or more diversity from this (i.e., between 75% to 89% id. over only 35% to 98% of the query sequence). There were no non-eukaryotic sequences in the top 100 Blast hits. As discussed below for the RAPD analysis, bacteria do potentially contaminate the isolates both internally and externally of the amoebae, but the related prokaryotic housekeeping gene homolog sequences show significantly lower identity—e.g., cytoskeletal homologs in bacteria share only 17–35% amino acid identity (5–11% nucleotide identity). Thus, these data give confidence that the genes identified were indeed of *N. perurans* origin.

In contrast to the MLST analysis, the RAPD analysis showed considerably more polymorphism among isolates. Each individual primer showed a unique pattern and, in turn, produced unique phylogeographic groupings. The combined results, however, were similar to those of the MLST analysis. Highly similar geographic patterns emerged but with poor support, supporting an ‘ubiquitous’ population. Geographical clustering patterns were observed in a microsatellite study on the global patterns of gene flow in a diatom *Pseudo-nitzschia pungens*, [35]. The authors suggested that this type of clustering pattern indicated that gene flow and migration rates were not strong enough to determine the sampling locations as one panmictic population [35]. So, though determined to be the same species, their data suggested that long-distance dispersal potentially occurred, but was not frequent enough to counteract the effects of population differentiation [35]. Even though *N. perurans* is not known to sexually reproduce, it is possible that a similar scenario occurs where dispersal/ migration is not strong enough to maintain a ‘global’ population. Therefore, we see this weak but consistent geographic pattern across both MLST and RAPD analyses.

Based on RAPD analyses, the *N. perurans* isolates appear to be less polymorphic than other species of amoebae, including *Acanthamoeba* spp. [26] and *Naegleria fowleri* [25], which used the same set of RAPD primers. Whether differences in the degree of polymorphism truly exist between these species is questionable, as the potential to identify polymorphism is directly related to the banding complexity generated from a primer and therefore affected by the reaction parameters specific to the study. Reaction components, such as different polymerases, primer concentrations, and thermal profiles, are all known to affect banding complexity, making it difficult to directly compare the degree of polymorphism and reproducibility between studies. This often-stated disadvantage of RAPD can nonetheless be addressed, as was the case in our study, by careful optimization of the assay that improves reproducibility, minimises PCR artifacts and maximises the ability to identify polymorphism. The RAPD results resembled the MLST pattern when all primers were combined. Similar to the *Naegleria fowleri* [25], the primer B10 was particularly useful in differentiating between geographic isolates, and showed the greatest similarity in phylogeographic pattern to the consensus pattern. In addition, the RAPD analysis allows for the exploration of bands linked to genes that are dissimilar between isolates. This is one of the major benefits of the RAPD analysis, especially for non-model organisms, and is of particular benefit in *N. perurans* due to the prolific nature of AGD and the lack of sequence data. Although further work is required to determine the origin of the RAPD band differences, the banding patterns are a starting point and indicate differences that were not visible with MLST. One area of interest for further research is the relationship between the parasome and amoebae nucleus, as it is not known how much the genetic material of the parasome influenced the RAPD results.

While the isolates were not axenic, the presence of bacteria did not confound the results, as shown by the qPCR quantification and culture supernatant RAPD comparisons. However, there is evidence that the presence of endosymbionts can cause population variation in RAPD analysis [36]. A study on population heterogeneity in the endoparasitoid of silkworms, the uzifly (*Exorista sorbillans*), in south India found that the populations were distinguishable by the type of *Wolbachia* endosymbiont the uzifly carried [36]. *Wolbachia* could be differentiated into supergroup A, B or a combination of A and B, which was used to determine geographic origin [36]. *Neoparamoeba’s* endosymbiont was incorporated into the genus from a single evolutionary endosymbiosis event and transferred vertically from mother to daughter in an obligate relationship [37]. As the parasome is obligate it would not be as discriminatory in influencing the RAPD analysis as *Wolbachia* is in uziflies. The Kinetoplastida group, from which the parasome originated, contains many disease-causing members, including known fish parasites such as *Trypanoplasma borreli*, an extracellular blood parasite of cyprinid fish [38], and *Ichthyobodo necator*, an ectoparasite of a wide range of fish species including salmon [39]. The role in which the parasome may influence virulence and contribute to the genetic diversity observed is not yet known, and, therefore, its influence cannot be discounted [8,40].

Clonal isolates, some of which have been shown to be avirulent after passage [41], were included in our analysis. Those clonal isolates showed a different RAPD pattern to those of non-clonal isolates obtained from the same location, which was particularly apparent in the banding patterns of primers B10, B12 and B18. The differences in patterns may be indicative of genetic changes in line with losing virulence. Previous studies using RAPD in parasitic or pathogenic eukaryotic species were shown to be useful in differentiating between highly virulent, less virulent and avirulent strains [42,43]. In particular, RAPD was useful for the crayfish fungal pathogen *Aphanomyces astaci*, where it was used first to differentiate between five genotypes based primarily on geography and then to show that new outbreaks were caused by a genotype not previously described [43].

The addition of more *N. perurans* isolates from AGD outbreaks and subsequent clonal sequences from (a) the countries compared in this study (b) other AGD affected countries (c) emergent outbreaks and (d) archival outbreaks would be particularly useful in resolving relationships between virulent *N. perurans* and inform both MLST and RAPD analysis. Both non-clonal and clonal samples should be considered. Virulence testing for all samples would add more information, but it was unfortunately not possible in this study, as most samples were obtained as DNA or fixed. Though environmental samples are ideal to get an accurate idea of the overall population, clonal samples help to resolve sequences where intragenomic variation occurs. It is envisaged that further work to incorporate more samples from existing AGD affected countries, as well as from new and archival AGD outbreaks, will further resolve potential genotypes and benefit the epidemiology of this important fish disease.

In conclusion, both MLST and RAPD identified small geographical differences between isolates, however these were insufficient to indicate strong geographical isolation and genetic drift, with no founder population identified. The data suggest that the increasing global trend in AGD outbreaks is not due to the spread of a particularly virulent *N. perurans* strain between geographical locations. Further work using a wider range of isolates from countries with AGD outbreaks would be beneficial to further resolve the genetic groupings observed and the epidemiology of the disease in these regions.

## 4. Materials and Methods

### 4.1. Strains and DNA Extraction

From Tasmania, isolates of *N. perurans* were cultured from Atlantic salmon (*Salmo salar*) sampled during AGD outbreaks on Atlantic salmon farms in the Huon Estuary, or reisolated from experimental challenges of Atlantic salmon in a 4000 L recirculating tank at 16 °C and 35 ppt. The amoebae to start the experimental challenges were also taken from infected gills from farms in the Huon Estuary. Clone 4 and Clone 8 were each established from a single amoeba isolated from amoebae cultures first harvested in 2008 from the challenge tanks outlined above. All samples were taken from cultures on 35 ppt seawater malt yeast agar plates incubated at 18 °C with a marine bacterial overlay. All samples were stored at -20 °C in RNA preservation solution (4 M ammonium sulphate, 25 mM sodium citrate, 10 mM EDTA; pH 5.2). In addition to samples from Tasmania, *N. perurans* isolates taken from farmed Atlantic salmon from Ireland, Scotland, Norway, the USA and Canada were sent in a variety of preservation solutions, lysis buffer (4 M urea, 1% SDS, 0.2 M NaCl, 1 mM sodium citrate), 96% ethanol and RNA preservation solution) and stored at -20 °C (Table 3).

DNA was extracted following a modified protocol Bridle et al. [41]. Briefly, the isolates stored in RNA preservation solution were spun at 16,000 g for 10 min to pellet the cells. This step appeared to be essential in recovering DNA from isolates stored in RNA preservation solution, perhaps due to a change in cell density from the high salt content of the solution. The supernatant was removed and the pellet was incubated with 500 µL lysis buffer (4 M urea, 1% SDS, 0.2 M NaCl and 1 mM sodium citrate) for 10 min at 55 °C with occasional vortexing. The tubes were then immediately placed on ice for 5 min, before the addition of 250 µL ammonium acetate (7.5 M). The tubes were vortexed for 20 s. The remaining steps were as described [41].

The isolates stored in lysis buffer were processed following the same protocol except that the initial pelleting step was omitted. The vials were vortexed to homogenize the mixture and 500 µL was removed and incubated at 55 °C for the specified 10 min. The gill samples stored in ethanol were processed using a slightly modified protocol. The largest quantity of *N. perurans* was found to be present in the ethanol used to store the gills rather than on the preserved gills. The DNA was then extracted from the ethanol preserved amoebae following the same protocol used for the RNA preservation solution samples.

### 4.2. MLST Loci

The eukaryotic MLST literature [12,16,48] was reviewed for suitable candidate housekeeping genes and cross referenced with Genbank sequences of *N. perurans* and other amoebae. Six genes were chosen [elongation factor 1 (*ef1*), elongation factor 2 (*ef2*), beta tubulin (*tubb*), beta-actin (*actb*), RNA polymerase large subunit 1(*Rpb1*), and succinate dehydrogenase complex flavoprotein subunit A (*sdha*)]. Gene fragments from 350 to 600 bp from each of the candidate genes were selected for DNA sequencing and homology-based oligonucleotide primers designed from available sequences in Genbank from *N. perurans* and/or related organisms (*Neoparamoeba pemaquidensis, Neoparamoeba brachiphila, Naegleria* spp., *Acanthamoeba* spp. *and Entamoeba* spp.). Sequences were aligned using the Geneious version 8.1.6 software [49] and three primer pairs were generated for each gene using Geneious primer prediction and manually adjusted for polymorphic nucleotide sites with a bias towards *N. pemaquidensis* sequences. All primers were then tested with *N. perurans* genomic DNA and the top primer pair for each gene chosen based on length, GC content, coverage of polymorphic sites, and suitability for direct DNA sequencing. The final sense and antisense primers are shown in Table 4.

An initial PCR reaction was carried out confirming *N. perurans* as the sole isolated *Neoparamoeba* spp. using primers for *N. perurans* [9], *N. pemaquidensis* [50] and *N. branchiphila* [6]. The 10 µL PCR reaction consisted of 5 µL 2× MyTaqHS mix (Bioline, NSW, Australia), 500 nM of each primer, 2 µL water and 2 µL template, following the amplification conditions: 3 min at 95 °C, 35 cycles of 30 s at 95 °C, 25 s at 55 °C and 10 s at 72 °C. The MLST PCR reactions were carried out in 20 µL reactions containing 10 µL 2× MyTaqHS mix (Bioline), 500 nM of each primer, 6 µL water and 2 µL genomic DNA template. Amplification conditions were as follows: an initial 3 min at 95 °C, then 15 s at 95 °C, 35 cycles of 30 s at [*ef2*—64.5 °C, *ef1*, *Rpb1*, *tubb*, *sdha*—58.4 °C and *actb* at 54.5 °C], 15 s at 72 °C, with a final extension of 1 min at 72 °C. Amplification products were assessed by size separation electrophoresis through agarose gel. In some instances (*Ppb1* and *actb*), where one single band could not be initially resolved, candidate bands were isolated by excision and gel purification and further amplified by an additional 25 PCR cycles using the same primers. Amplified products were purified using SureClean plus (Bioline) and directly sequenced in both directions (Macrogen, Seoul, Korea). Primers used in both sense and antisense sequencing were identical to primers used for amplification (Table 4).

### 4.3. MLST Data Analysis

Alignments were created for each individual gene fragment with the 16 selected isolates using the Geneious alignment software and heterozygotes were identified using a peak similarity threshold of 90% to determine “real” heterozygotic and ambiguous sites. The forward and reverse sequences were then compared for each isolate to confirm the validity of the sequences.

The MLST data were analysed using the MLSTest software [20] with the objective of identifying geographic subtypes based on nucleotide diversity. Allelic profiles were created, in which the MLSTest software calculated the Typing Efficiency (TE) for each allele. The TE is a representation of the number of identified genotypes within a gene divided by the number of polymorphic sites. The discriminatory power, or the probability that two strains can be differentiated based on that gene when pulled at random from a population, was also calculated using the MLSTest software [51].

### 4.4. RAPD PCR Amplification

Five 10 nucleotide primers were chosen: A1 (5′CAGGCCCTC3′), A15 (5′TTCCGAACCC3′), B10 (5′CTGCTGGGAC3′), B12 (5′CCTTGACGCA3′) and B18 (5′CCACAGCAGT3′). These primers were previously reported in a study on the free-living amoeba *Naegleria fowleri* [25]. Each PCR reaction mixture was made using MyTaq HS mix (Bioline), which consists of a propriety buffer containing preoptimized MgCl_2_ and dNTPs, therefore these parameters were not taken into consideration. The final reaction volume was 20 µL and consisted of 10 µL 2× MyTaq HS, 1 µL of primer (10 µM), 7 µL distilled water and 2 µL DNA template. The initial PCR was run using the following protocol: an initial 2 min at 95 °C, 35 cycles of 15 s at 95 °C, 30 s at 40 °C, 15 s at 72 °C, with a final extension of 1 min at 72 °C. Following the initial PCR, a 1 in 10 dilution was made from each set of PCR products using distilled water. These diluted products were then amplified for a further 30 cycles. Each primer PCR reaction was run in triplicate to gauge reproducibility. The PCR products were size-separated by electrophoresis on a 1.2% agarose gels stained with RedSafe (iNtRON Biotechonolgy) using a 1kb molecular ladder.

### 4.5. PhyElph Analysis

Dendrograms of the relatedness of the strains were created using the PyElph program [52]. The gels from all three replicates for each primer were visually compared to each other and a representative gel image for each primer was uploaded into the program. The PyElph program detected the lanes, migration bands and molecular weights; manual corrections were made where necessary. A similarity matrix was created though the program using the dice coefficient and then used to construct a neighbour joining dendrogram for each primer (Figure 1).

### 4.6. Statistical Analysis

A Jaccard coefficient distance matrix was constructed for the combined primer profiles, generated during the RAPD approach, by marking the presence or absence of a band as 1/0. Similarity and the inverse dissimilarity for each isolate was computed using the XLSTAT add on for Microsoft Excel [53]. The dissimilarity matrix was then inputted into the Trex—online web program under the neighbour joining option taken from Saitou and Nei [27] and a dendrogram of the data was produced [54].

In relation to the MLST analyses, concatenated neighbour joining trees were created using both average states and SNP duplication to address heterozygous sites [20]. Overall phylogenetic incongruences were addressed using the incongruence length difference test using the BIO-Neighbour joining method (BIONJ-ILD) with 1000 replications, which is used to determine whether one or multiple fragments support a phylogeny that is not supported by the remaining fragments (P < 0.001).

A basic BURST (Based upon related sequence types) analysis was run with the MLSTest program using a group definition of five shared alleles. The BURST and tree analysis are based on the concatenation of alignments [20].

## Figures and Tables

**Table 1:** *N. perurans* MLST targets showing the Typing Efficiency (TE) and Discriminatory Power (DP) for each gene loci (elongation factor 1 (*ef1*), beta tubulin (*tubb*), RNA polymerase large subunit 1 (*Rpb1*), beta actin (*actb*), elongation factor 2 (*ef2*) and succinate dehydrogenase complex flavoprotein subunit A (*sdha*), along with the relative variability of each gene.

Gene Loci	No. of Genotypes	No. of Polymorphic Sites	Typing Efficiency	Discriminatory Power	Genbank Acquisition Number
***actb***	2	1	2	0.588	KX363877 KX363880
***tubb***	2	1	2	0.228	KX363876 KX363879
***ef1***	1	0	infinite	0.118	KX363881
***ef2***	3	1	3	0.551	KX363875 KX363882 KX363878
***Rpb1***	1	0	Infinite	0.118	KX363883
***sdha***	1	0	Infinite	0.118	MN399678

**Table 2:** Jaccard coefficient similarity distance matrix for the combined RAPD primer profiles (A1, A15, B10, B12 and B18).

	Tas C8	Tas C4a	Tas C4b	Tas C4c	Tas C4d	Tas 1	Tas 2	Tas 3	Norway	Canada	USA	Ireland 1	Ireland 2	Scotland 1 C	Scotland 2 1	Scotland C 2
Tas C8a	1															
Tas C4a	0.667	1														
Tas C4b	0.806	0.719	1													
Tas C4c	0.667	0.742	0.774	1												
Tas C4d	0.576	0.759	0.625	0.700	1											
Tas 1	0.195	0.231	0.225	0.231	0.216	1										
Tas 2	0.167	0.200	0.195	0.200	0.184	0.680	1									
Tas 3	0.171	0.175	0.200	0.205	0.158	0.708	0.640	1								
Norway	0.250	0.289	0.282	0.324	0.353	0.265	0.229	0.200	1							
Canada	0.293	0.300	0.325	0.368	0.289	0.243	0.243	0.216	0.516	1						
USA	0.149	0.128	0.174	0.152	0.111	0.343	0.343	0.278	0.200	0.342	1					
Ireland 1	0.111	0.089	0.111	0.114	0.070	0.132	0.162	0.105	0.158	0.205	0.297	1				
Ireland 2	0.220	0.195	0.190	0.167	0.211	0.194	0.194	0.200	0.189	0.205	0.333	0.189	1			
Scotland C	0.179	0.250	0.211	0.216	0.235	0.114	0.147	0.152	0.111	0.162	0.128	0.143	0.176	1		
Scotland 1	0.179	0.154	0.179	0.154	0.200	0.219	0.147	0.188	0.212	0.162	0.158	0.176	0.290	0.091	1	
Scotland 2	0.179	0.154	0.179	0.154	0.200	0.219	0.147	0.188	0.212	0.162	0.158	0.176	0.290	0.091	1	1

**Table 3:** *Neoparamoeba perurans* isolates from Atlantic salmon listed by country of origin, isolates name, year of isolation, the type/source of culture, the type of fixation used (‘lab isolates’ indicate isolates that were not preserved before DNA extraction), and the year that amoebic gill disease (AGD) outbreaks were first recorded for that country in the literature.

Origin	Isolate	Year	Type	Fixation	Year of First Recorded AGD Outbreak (Reference)
**Tasmania, Australia**	Tasmania C8a	First Isolated 2008, In clonal culture since 2010	Clonal	Lab Isolate	1985 [3]
**Tasmania, Australia**	Tasmania C4a	First Isolated 2008, In clonal culture since 2010	Clonal	Lab Isolate	
**Tasmania, Australia**	Tasmania C4b	First Isolated 2008, In clonal culture since 2010	Clonal	Lab Isolate	
**Tasmania, Australia**	Tasmania C4c	First Isolated 2008, In clonal culture since 2010	Clonal	Lab Isolate	
**Tasmania, Australia**	Tasmania C4d	First Isolated 2008, In clonal culture since 2010	Clonal	Lab Isolate	
**Tasmania, Australia**	Tasmania 1	2014		RNA preservation solution	
**Tasmania, Australia**	Tasmania 2	2015		RNA preservation solution	
**Tasmania, Australia**	Tasmania 3	2015		RNA preservation solution	
**Ireland**	Ireland 1	2014		RNA preservation solution	1995 [44]
**Ireland**	Ireland 2	2015		RNA preservation solution	
**Scotland**	Scotland 1	2012		RNA preservation solution	2006 [45]
**Scotland**	Scotland 2	2014		RNA preservation solution	
**Scotland**	Scotland C	2014	Clonal	RNA preservation solution	
**Washington, USA**	USA	2015	Mixed Gill Isolation	Lysis Buffer	1985 [4]
**British Columbia, Canada**	Canada	2015	Mixed Gill Isolation	Lysis Buffer	2014 [46]
**Norway**	Norway	2014	Gill Arch	96% Ethanol	2006 [47]

**Table 4:** MLST genes: elongation factor 1 (*ef1*), beta tubulin (*tubb*), RNA polymerase large subunit 1 (*Rpb1*), beta actin (*actb*), elongation factor 2 (*ef2*) and succinate dehydrogenase complex flavoprotein subunit A (*sdha*) with the primer sequences and amplicon length of the fragments used in the analysis.

Gene	Sense Primer Sequence 5′-3	Antisense Primer Sequence	Amplicon Length (bp)
***ef1* Sense**	AGAAGGAAGCCGCCGATATG	GACAACCATACCGGGCTTCA	558
***ef2* Sense**	GAGGAGTACGCCCAAATCCC	CCATAGATACCACCACGGGC	480
***Rpb1* Sense**	GCTGAGGATCGACCCCAAAA	CGCGACGTATCTCTGAAGCT	512
***bact* Sense**	CATCTATGAGGGTTATG	GATGATCTTGATCTTCA	375
***tubb* Sense**	CTTTGTCCCTCCACCAGCTT	CGCTGGACTTTTGTTGGAGC	378
***sdha* Sense**	GGTGGTATTACTGGACGACATCT	GGCAGAGATTGGAAGGAA	340
